# Multiple Cranial Nerve Palsies in a Pediatric Case of Lemierre's Syndrome due to *Streptococcus viridans*

**DOI:** 10.1155/2021/4455789

**Published:** 2021-10-26

**Authors:** Samantha Novotny, Kenneth Serrano, Danielle Bazer, Louis Manganas

**Affiliations:** ^1^Renaissance School of Medicine at Stony Brook University, 101 Nicolls Road, Stony Brook, New York 11794, USA; ^2^Department of Neurology at Stony Brook University Hospital, 101 Nicolls Road Stony Brook, New York 11794, USA

## Abstract

**Background:**

Lemierre's syndrome is a rare condition of internal jugular vein thrombosis following oropharyngeal infection. While it usually results from *Fusobacterium necrophorum* infection, atypical cases associated with other pathogens have been reported.

**Objective:**

To describe a unique case of pediatric Lemierre's syndrome with *Streptococcus viridans* infection resulting in cavernous sinus thrombosis and oculomotor, trochlear, and abducens nerve palsies. *Case Report*. A 14-year-old female initially presented after six days of fever, myalgias, and sore throat and was admitted for hyperbilirubinemia and acute kidney injury. She developed a fixed, dilated pupil with complete ophthalmoplegia, ptosis, and severe pain. Imaging revealed retromandibular space abscess, external and internal jugular vein thrombosis, cavernous sinus thrombosis, internal carotid artery stenosis, pulmonary embolism, and bilateral pneumonia. She was diagnosed with Lemierre's syndrome with cultures positive for *Streptococcus viridans* and treated with a combination of antibiotics and anticoagulation. *Conclusion and Relevance*. Both antibiotics and anticoagulation were effective management for this Lemierre's syndrome patient with cavernous sinus thrombosis. Early diagnosis and treatment of Lemierre's syndrome is essential. A multidisciplinary treatment team is beneficial for managing the sequelae of this condition.

## 1. Introduction 

Lemierre's syndrome is a rare sequela of oropharyngeal infection in previously healthy young adults resulting in thrombosis of the internal jugular vein and septic embolism [1]. The most common offending organism is *Fusobacterium necrophorum* [2], although other organisms have been reported including *Staphylococcus aureus* and several strains of *Streptococcus* [3–9]. We describe a unique case of pediatric Lemierre's syndrome caused by *Streptococcus viridans* with multiorgan involvement and progression to cavernous sinus thrombosis with cranial nerves III, IV, and VI palsies. While Lemierre's syndrome associated with *Streptococcus viridans* infection has previously been described, our patient with development of complete ophthalmoplegia demonstrates an underreported sequela of this condition.

## 2. Case Presentation

A 14-year-old female with no medical history presented to the emergency department with a six-day history of fever, myalgias, pharyngitis, throbbing headache, and left eye ptosis. Her symptoms were associated with diplopia, blurred vision, photophobia, and phonophobia. Her neurological exam was significant for visual acuity of 20/200 in the left eye with an afferent pupillary defect. There were also cranial nerve III, IV, and VI palsies.

**Figure 1:**
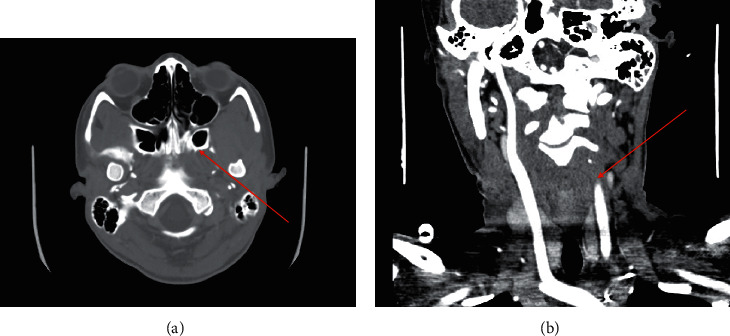
CT angiography. (a) CT angiography venous phase of the head, demonstrating left CST. (b) CT angiography head demonstrating left ICA occlusion.

Throat swab cultures grew *Candida dubliniensis* and viridans group alpha hemolytic *Streptococcus*. Her hospital course was complicated by left retromandibular space abscess, left external jugular vein thrombosis, partial left internal jugular vein thrombosis, bilateral lower lobe pneumonia, and multiple pulmonary consolidations with wedge-shaped defects in the right upper lobe consistent with embolism. Additionally, compute tomography (CT) angiography head, magnetic resonance imaging (MRI) brain, magnetic resonance angiography (MRA) head and neck, and magnetic resonance venography (MRV) head and neck revealed left cavernous sinus thrombosis (CST) with adjacent pachymeningitis, possible cavernous sinus abscess, severe stenosis of the left distal petrous and cavernous internal carotid artery (ICA), focus of severe stenosis in the ICA terminus, and dural thickening and enhancement (Figure 1). The patient underwent two cerebral angiograms; the initial showed left ICA dissecting aneurysm with stenosis with posterior communicating artery collateral flow. The second angiogram four days later showed a complete, left ICA thrombosis.

**Figure 2:**
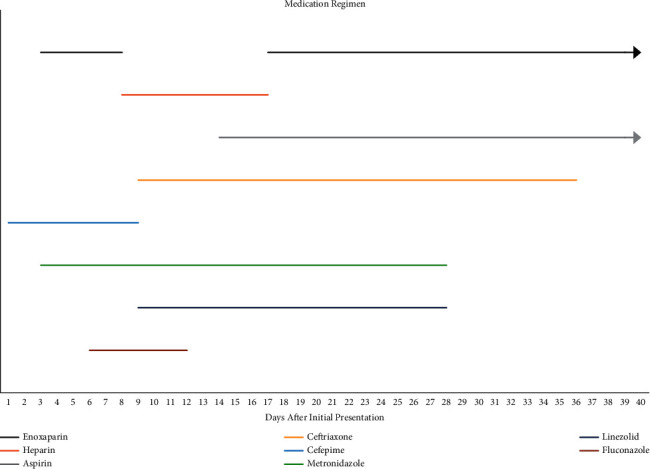
Timeline of the patient's medication regimen.

The patient was managed with a medication regimen including both antibiotics and anticoagulation with age and weight-appropriate dosing. Her medication timeline is summarized in Figure 2. She was managed by various specialties including neurology, neurosurgery, ophthalmology, infectious disease, and hematology. The neurosurgery team determined that medical management without surgical intervention was sufficient. She remained on aspirin and enoxaparin after discharge. Unfortunately, her neurological deficits were persistent upon discharge home with outpatient follow-up. Repeat imaging 28 days after initial presentation revealed worsened left ICA occlusion and significant partial resolution of the cavernous sinus thrombosis.

## 3. Discussion

We describe a young female with cavernous sinus thrombosis and multiple cranial nerve palsies resulting from Lemierre's syndrome associated with *Streptococcus viridans*. Lemierre's syndrome is uncommon, particularly with organisms other than Fusobacteria [10]. A review by Riordan [10] suggests merely 8% of Lemierre's syndrome cases are due to other pathogens [10]. However, among patients with ophthalmic symptoms, 25% had *Streptococcus* infections [5]. Reports of Lemierre's syndrome with various strains of *Streptococcus* have described complications including CST and isolated cranial nerve palsies, as well as other findings including postseptal cellulitis, pulmonary embolism, pleural effusion, and bibasilar pneumonia [5–9]. While some of these case reports noted isolated trochlear or abducens nerve palsies, they did not report any instances of multiple cranial nerve palsies.

Lemierre's syndrome and its complications can be life-threatening [10, 11]. Thus, prompt diagnosis and treatment of the condition are essential. However, there is no clear consensus on treatment for Lemierre's syndrome. Management is typically focused on treating the infection via antibiotics and drainage as necessary [12]. Anticoagulative therapy is controversial. Riordan [10] suggested around 23% of patients receive anticoagulation [10], while a more current review by Johannesen and Bodtger [13] found up to 64% are treated with anticoagulation [13]. A systematic review by Adedeji et al. [14] studied 14 patients and found anticoagulation to be a safe therapeutic strategy in Lemierre's syndrome [14]. However, Gore [11] performed a meta-analysis that found anticoagulation did not significantly impact recanalization of thrombosed vessels or mortality rates [11]. Despite this, it has been suggested that decision to anticoagulate may be patient-specific. A review by Kristensen and Prag [15] recommended anticoagulation should only be given in patients at risk of thrombosis extension to the cavernous sinus [15]. Similarly, another review by Moore et al. [16] of 41 patients found 26.8% of patients improved with addition of anticoagulation and proposed use of anticoagulation in patients with extensive thrombosis [16]. For our patient, anticoagulation was initiated during hospitalization and continued after discharge.

## 4. Conclusion

We describe a pediatric case of Lemierre's syndrome caused by an unusual pathogen, *Streptococcus viridans.* Our patient developed extensive thrombosis, multiorgan involvement, and unique sequelae of cranial nerves III, IV, and VI palsies. She was treated on a regimen of antibiotics and anticoagulation. This case highlights the importance of a multidisciplinary, team-based approach to patient care. Rapid diagnosis of this rare syndrome is crucial for proper therapeutic management. It is beneficial for neurologists to be aware of Lemierre's syndrome, as there are potentially neurologically devastating sequelae of the syndrome. Moreover, future research and randomized controlled trials are warranted to develop consensus guidelines for anticoagulation and treatment of Lemierre's disease.

## Data Availability

Data for this article were gathered from the patient's medical record.
